# Genotoxicity, DNA damage and sperm defects induced by vinblastine

**DOI:** 10.1007/s11033-022-08061-1

**Published:** 2022-11-17

**Authors:** Maha A. Fahmy, Entesar E. Hassan, Ayman A. Farghaly, Zeinab M. Hassan

**Affiliations:** 1 Department of Genetics and Cytology, National Research Centre (NRC), Dokki, Giza, Egypt; 2 Department of Natural Compounds Chemistry Research, National Research Centre (NRC), Dokki, Giza, Egypt

**Keywords:** Vinblastine, Chromosomal aberrations, Bone marrow, Spermatocytes, DNA damage, Sperm abnormalities

## Abstract

**Background:**

The treatment with chemotherapy may develop secondary tumors as a result of chemo genotoxicity. Sperm defects is another complication associated with chemo treatment. In this study the genotoxicity of vinblastine (VB) was estimated in both somatic and germ cells.

**Materials:**

85 mice were taken. Four single doses of VB at 3, 4.5, 6 and 10 mg/kg and three successive doses at 3, 4.5 and 6 mg/kg were taken for estimation of chromosomal aberrations (CAs). Four single doses of VB were involved in estimating the DNA fragmentation, and comet assay. For sperm abnormalities mice were injected with three successive doses of VB at 3, 4.5, and 6 mg/kg.

**Results:**

The results demonstrated a significant frequency of DNA fragmentation in spleen cells and in the percentage of CAs in bone marrow. Numerical and structural aberrations were recorded with a pronounced number of polyploidy metaphases which reached (11.60%) after treatment with 6 mg/kg for three successive days vs zero for control. VB also induced a significant percentage of CAs in spermatocytes in the form of univalent. Sperm defects in the form of coiled tail, absence of acrosome and shapeless head and a significant DNA damage in the testes were recorded. The frequency of sperm abnormalities reached 11.06 ± 0.14 after treatment with highest tested dose (6 mg/kg) vs 3.04 ± 0.19 for control.

**Conclusion:**

VB is genotoxic in somatic and germ cells. Sperm defects induced by VB are of serious concern to future generations and may affect the fertility of cancer survivors.

## Introduction

Vinblastine (VB) is a very important clinic alkaloid extracted naturally from a periwinkle plant which is a flowering herb from *Cathar anthusroseus* [1]. It is a chemotherapeutic medication used separately or in combination with other cytotoxic drugs (bleomycin, doxorubicin, and methotrexate…) for the treatment of a wide range of human neoplasms e.g. Hodgkin and non-Hodgkin’s lymphoma, T-cell lymphoma, testicular and germ cell tumors, bladder, lung (Non-small cell lung cancer), head and neck cancers. It is also used to treat melanoma, soft tissue sarcoma, Kaposi’s sarcoma, gestational trophoblastic and some blood disorders (e.g. histiocytosis X) [2, 3]. In some cases it exerts an immunosuppressive effects. VB is one of the World Health Organization List of Essential Medications. It is a member of the Vinca alkaloid family which are anticancer agents that include the natural product vinblastine, vincristine, and the semi-synthetic product vin-orelbine [4]. Vinca alkaloids have the potential to induce cytotoxic and genotoxic effects, oxidative DNA damage and micronuclei formation [2, 5]. Vinca alkaloids exert their function as mitotic spindle inhibitors [6].They are antimitotic drugs that inhibit microtubules and are cell cycle phase-specific ‘S and M phase poisons’ [7]. VB is potentially mutagenic and animal studies have demonstrated degenerative changes in germ cells. Palo et al. [8] reported that vinblastine is cytogenotoxic to mouse spermatogonia and that such induced effects are transmissible in the male germline cells of Swiss mice. Also AL-Zubaidi [9] reported that different doses (4, 5 and 6 mg/kg) of VB induced a significant decrease (P < 0.05) in the mice sperm concentration, sperm viability percentage and sperm motility (p < 0.001) compared to control.

A major problem with cancer chemotherapy is its severe toxic effect on non-target tissues (normal cells), so the cancer patients not only suffer from an immediate side effects of cancer medications but are also subjected to an increased risk of developing secondary tumors later in life [10]. Recent studies in chemotherapy approaches claim at developing medications that eradicate cancer cells efficiently and selectively. So it is of special need to study the side effects of anticancer agents in order to improve the conventional chemotherapy by protecting healthy tissues. In the present work, a comprehensive study was performed to determine the genotoxicity and DNA damage induced by the anticancer drug vinblastine. Not only one technique can determine the genotoxic effect of chemicals. Previous studies did not including all these cytogenetics parameters and estimated one or two parameters only. Previous studies were inconclusive and controversial. Also in the present study, a wide range of doses was estimated, which may cover all the doses required for treating different types of human malignancies. This study also included a comparison of the genotoxic effects of vinblastine in somatic and germ cells. It gave details about different types of sperm abnormalities.

## Materials and methods

### Chemicals

Common trade name of the chemotherapeutic drug is vinblastine sulfate, velban and others. Vinblastine was obtained from Sigma Aldrich (MO, USA). Drug Bank Accession Number: DB00570. All other chemicals were purchased from ADWICC (Cairo, Egypt).

### Animals

Male white Swiss mice (*Mus musculus*), aged 9 to 12 weeks, were used in all experiments. Animals were obtained from a closed random-bred colony at the National Research Centre (Giza, Egypt). Mice used for each experiment were of similar age (± 1 week) and weight (± 2 g). Animals were housed in polycarbonate boxes with steel wire tops and bedded with wood shavings. Ambient temperature was controlled at 22 ± 3 °C with a relative humidity of 50 ± 15% and a 12 h light/dark photoperiod. Food and water were provided ad libitum. The experiments were conducted according to the Animal Research Ethical Committee Guidelines of the National Research Centre, Egypt. The Approval Certificate is under number: 19 163.

### Experimental design

In these experiments a total of 85 mice were taken as follows: 40 mice for chromosomal aberrations in bone marrow and mouse spermatocytes, 25 mice for DNA fragmentation and alkaline comet assay and 20 for morphological sperm abnormalities. In each of these testes, mice were subdivided into groups (5/group).

The main groups represented chromosomal aberration analysis: Group I: Negative control, Groups II–V, mice were i.p injected with a single dose of VB (3, 4.5, 6 and 10 mg/kg), Groups VI–VIII in which mice received repeated i.p injections (three successive days) of VB at the dose levels 3, 4.5 and 6 mg/kg. In all experiments samples were taken 24 h after the last treatment.

Analysis for DNA damage, showed five groups: Animals were treated with a single i.p injection of VB at the dose levels 3, 4. 5, 6 and 10 mg/kg, in addition to the control group. Samples were taken 24 h after injection.

Analysis of sperm abnormalities showed four groups as follows: Negative control group and three treated groups with VB (3, 4.5 and 6 mg/kg, 3 injections) and samples were taken 35 days after the 1st injection. Different doses were taken to cover all doses required for different types of human cancerous and as previous reports concerning the doses of VB [9, 11].

### Cytogenetic analysis

#### Chromosomal aberration assay in mouse bone marrow

Mitotic and meiotic chromosomes were prepared from bone marrow and testis of the same animal, respectively. Bone marrow chromosomes were prepared according to the technique described by Fahmy et al. [12]. In brief, mouse bone-marrow cells were collected from both femurs, cells were incubated in hypotonic solution (KCL 0.075 M) for 20 min at 37 °C, and then centrifuged. The cell pellets were suspended in a fixative (methanol/glacial acetic acid 3:1). This step was repeated at least twice, then the cells were suspended in a few drops of fixative and spread onto frozen slides, air-dried, stained with 10% Giemsa for 30 min, washed, and air dried again. One hundred well-spread metaphases were analyzed per mouse describing different kinds of chromosome abnormalities (CAs) in bone marrow cells. Scoring for CAs was performed under 2000 × magnification with a light microscope.

#### DNA fragmentation assay in mouse spleen cells

##### DNA gel electrophoresis laddering assay

Apoptotic DNA fragmentation was qualitatively analyzed by detecting the laddering pattern of nuclear DNA as described according to Lu et al. [13]. Briefly, spleen tissues were homogenized, washed in PBS, and lysed in 0.5 ml of DNA extraction buffer (50 mM Tris–HCl, 10 mM EDTA. 0.5% Triton, and 100 μg/ml proteinase K, pH 8.0) for overnight at 37 °C. The lysate was then incubated with100 μg/ml DNase-free RNase for 2 h at 37 °C, followed by three extractions of an equal volume of phenol/chloroform (1:1 v/v) and a subsequent re-extraction with chloroform by centrifuging at 15,000 rpm for 5 min at 4 °C. The extracted DNA was precipitated in two volume of ice-cold 100% ethanol with 1/10 volume of 3 M sodium acetate, pH 5.2 at − 20 °C for 1 h, followed by centrifuging at 15,000 rpm for 15 min at 4 °C. After washing with 70% ethanol, the DNA pellet was air-dried and dissolved in 10 mM Tris–HCl/1 mM EDTA, pH 8.0. The DNA was then electrophoresed on 1.5% agarose gel and stained with ethidium bromide in Tris/acetate/EDTA (TAE) buffer (pH 8.5, 2 mM EDTA, and 40 mM Tris–acetate). A 100-bp DNA ladder (Invitrogen, USA) was included as a molecular size marker and DNA fragments were visualized and photographed by exposing the gels to ultraviolet trans-illumination.

##### Diphenylamine reaction procedure

Animal spleen tissues were used to determine the quantitative profile of the DNA fragmentation. Spleen samples were collected immediately after sacrificing the animals. The tissues were lysed in 0.5 ml of lysis buffer containing, 10 mM tris–HCl (pH 8), 1 mM EDTA, 0.2% triton X-100, centrifuged at 10 000 rpm (Eppendorf) for 20 min at 4 °C. The pellets were re-suspended in 0.5 ml of lysis buffer. To the pellets (P) and the supernatants (S), 0.5 ml of 25% tri-chloroacetic acid (TCA) was added and incubated at 4 °C for 24 h. The samples were then centrifuged for 20 min at 10,000 rpm (Eppendorf) at 4 °C and the pellets were suspended in 80 ml of 5% TCA, followed by incubation at 83 °C for 20 min. Subsequently, to each sample 160 ml of Diphenyl Amine (DPA) solution [150 mg DPA in 10 ml glacial acetic acid, 150 ml of sulfuric acid and 50 ml acetaldehyde (16 mg:ml) was added and incubated at room temperature for 24 h [14]. The proportion of fragmented DNA was calculated from absorbance reading at 600 nm wavelength using the formula:

$$\% {\text{ Fragmented DNA }} = \, \left[ {{\text{OD}}\left( {\text{S}} \right)/} \right[{\text{OD}}\left( {\text{S}} \right) \, + {\text{ OD}}\left( {\text{P}} \right)] \, \times \, 100$$

(OD: optical density, S: supernatants, P: pellets).

### Comet Assay in the testes

Comet assay was performed referring to the protocol developed by Blasiak et al. [15] with minor modifications. Cells from testes of each treatment were mixed with low-melting-point agarose (ratio of 1:10 v/v), then pipetted to precoated slides with normal-melting-point agarose. The slides were kept flat at 4 °C for 30 min in dark environment. The third layer of low melting point agarose was then pipetted on slides, left to solidify for 30 min at 4 °C. The slides were transferred to pre-chilled lysis solution, kept for 60 min at 4 °C. After that, slides were immersed in freshly prepared alkaline unwinding solution at room temperature in the dark for 60 min. Slides were subjected to an electrophoresis run at 0.8 V/cm, 300 mAmps at 4 °C for 30 min. The slides were rinsed in neutralizing solution followed by immersion in 70% ethanol and then air-dried. Ethidium bromide was used for slides stain and then visualized by using Zeiss epifluorescence microscope (510–560 nm, barrier filter 590 nm) with a magnification of × 400. 100 cells per animal were scored then analyzed with DNA damage analysis software (Comet Score, TriTek corp., Sumerduck, VA22742). The non-overlapping cells were randomly selected and were visually assigned a score on an arbitrary scale of 0–3 (i.e., class 0 = no detectable DNA damage and no tail; class 1 = tail with a length less than the diameter of the nucleus; class 2 = tail with length between 1 × and 2 × the nuclear diameter; and class 3 = tail longer than 2 × the diameter of the nucleus) based on perceived comet tail length migration and relative proportion of DNA in the nucleus [16, 17].

### Chromosomal aberration assay in mouse spermatocytes

Spermatocyte chromosomes were prepared from the testes according to the protocol described by Evans et al. [18] with some modifications [19]. Briefly, the testis was removed and squashed into a petri dish containing an isotonic solution of 2.2% trisodium citrate. Then the cell suspension was centrifuged for 5 min at 1500 rpm. The cell pellet was incubated in a hypotonic solution of 1.1% trisodium citrate for 20 min at 37 °C followed by centrifugation. The cell pellet was washed twice by a freshly prepared fixative. A few drops of the fixative cell suspension were dropped in clean microscopic slides, air dried and stained with 10% Giemsa stain. One hundred well-spread metaphases using light microscope under 2000 × magnification were analyzed per mouse describing different kinds of chromosome abnormalities (CAs) in mouse spermatocytes.

### Sperm shape abnormalities

Sperm were prepared according to the recommended method of Wyrobek and Bruce [20] with some modifications recorded by Fahmy et al. [21] and smears were stained with 1% Eosin Y. A total of 1000 sperm were counted per animal (5000/each treatment), and different types of sperm abnormalities were scored (Head & Tail abnormalities). Sperm preparations were examined by light microscopy at 1000 × magnification.

### Data analysis

Data were analyzed using computerized software SPSS (Statistical Package of Social Science, version 20, Armonk, New York: IBM Corp). The data were checked for normality and the homogeneity of the variance using the Kolmogorov–Smirnov’s test and Levene’s test, respectively. The differences among groups with normal distribution were analyzed by one-way analysis of variance (ANOVA) followed by the Tukey HSD test. The results were regarded as significant when the P-value was less than or equal to 0.05.

## Results

### Chromosomal aberration analysis in bone marrow cells

(Table 1) presents the frequency of chromosomal aberrations (CAs) induced in mice bone marrow cells after treatment with VB. The results indicated a significant percentage of CAs with a dose-related relationship in both single and repeated treatments. The percentage of aberrations reached 12.0 ± 0.95 and 19.40 ± 0.56 after single treatment with the dose 10 mg/kg and repeated treatment with the dose 6 mg/kg respectively vs 3.20 ± 0.37 for the control. Repeated treatment with VB recorded higher percentage of aberrations compared to the single dose treatment e.g. the values of CAs reached 10.20% vs 19. 40% for single and repeated treatment with the dose 6 mg/kg VB respectively.

**Table 1:** Frequency of chromosomal aberrations induced in mouse bone marrow cells after treatment with vinblastine

Treatment and doses	Total abnormal metaphases		No and (%) of metaphases with different types of chromosome aberrations		
	No	Mean(%) ± SE	Gap	Fragment and/or Break	Polyploidy
I. Control (Non-treated)	16	3.20 ± 0.37^a^	7(1.40)	9(1.80)	–
II. Vinblastine a-single dose					
3 mg/kg	32	6.40 ± 0.60^b^	6(1.20)	10(2.0)	16(3.20)
4.5 mg/kg	41	8.20 ± 0.58^b,c^	5(1.0)	12(2.40)	24(4.80)
6 mg/kg	51	10.20 ± 0.37^c,d^	6(1.20)	20(4.0)	25(5.0)
10 mg /kg	60	12.0 ± 0.95^d,e^	8(1.60)	13(2.60)	39(7.80)
b-3 successive dose treatment					
3 mg/kg	65	13.0 ± 0.95^e^	8(1.60)	12(2.40)	45(9.0)
4.5 mg/kg	83	16.60 ± 0.93^f^	11(2.20)	22(4.40)	50(10.0)
6 mg/kg	97	19.40 ± 0.56^g^	14(2.80)	25(5.0)	58(11.60)

A total of 500 cells were analyzed (five mice per group; 100 cells/mouse). One way ANOVA–Tukey’s multiple comparisons test was used. The values having different superscript letters in each column are significantly different from one another

With respect to the types of aberrations polyploidy metaphases represented the major percentage of aberrant cells followed by breakage and gap. The frequency of polyploidy reached 7.8 and 11.6% of the total counted metaphases after treatment with a single dose of 10 mg/kg and repeated doses of 6 mg/kg VB respectively.

### DNA damage in spleen tissues

**Fig. 1:**
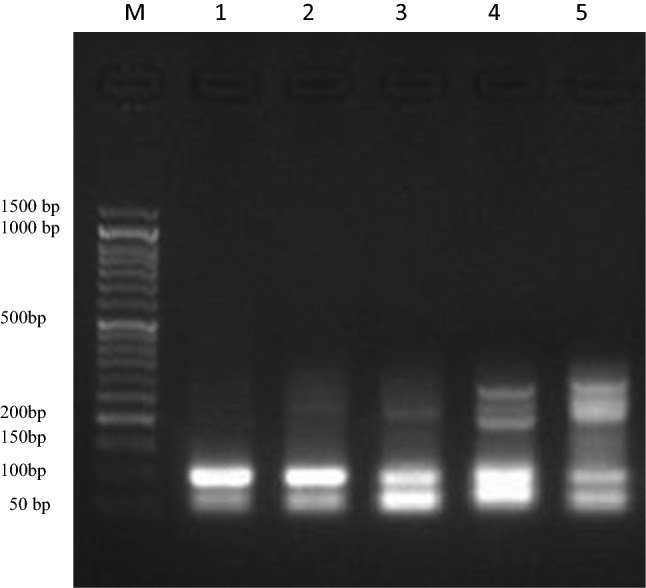
DNA fragmentation detected with agarose gel of DNA extracted from spleen tissues of mice exposed to different doses of vinblastine. Lane M represents DNA marker. Lane 1 represents negative control group. Lane 2, 3, 4 and 5 represent mice treated with the doses 3, 4.5, 6, and 10 mg/kg vinblastine respectively

Rate of DNA fragmentation in spleen tissues of male mice exposed to different doses of vinblastine (3, 4.5, 6 and 10 mg) is presented in Table 2 and Fig. 1. The results revealed that the dose 3 mg/kg VB had relatively similar rate of DNA fragmentation compared to control group (10.2% vs 9.5% respectively). In contrast, the DNA fragmentation rate values were increased significantly and dose-dependent in spleen samples of mice exposed to other doses of VB compared to control group. Moreover, male mice exposed to 6 and 10 mg of VB exhibited higher DNA fragmentation (18.6 and 24.2%) compared to those exposed to 3 and 4.5 mg/kg VB (10.2 and 12.4% respectively). The highest DNA fragmentation was found in mice exposed to 10 mg/kg VB.

**Table 2:** DNA fragmentation in spleen cells and comet analysis in the testes of male mice exposed to different doses of vinblastine

Treatment and doses	DNA Fragmentation % in spleen (Mean ± SE)	No. of cells with comets in testes	Class*				DNA damaged cells % (Mean ± SE)
			0	1	2	3	
(−ve) control	9.5 ± 0.71^a^	36	464	25	11	0	7.21 ± 1.07^a^
Vinblastine 3 mg	10.2 ± 0.49^a,b^	39	461	23	16	0	7.83 ± 0.93^b^
4.5 mg	12.4 ± 0.47^c^	51	449	28	17	6	10.24 ± 1.24^c^
6 mg	18.6 ± 0.67^c,d^	83	417	31	28	24	16.61 ± 1.50^c,d^
10 mg	24.2 ± 0.88^d^	107	393	35	31	41	21.43 ± 1.21^d^

A total of 500 cells were analyzed for comets (five mice per group; 100 cells/mouse). *: Class 0 = no tail; 1 = tail length < diameter of nucleus; 2 = tail length between 1 × , and 2 × the diameter of nucleus; and 3 = tail length > 2 × the diameter of nucleus. Data are presented as Mean ± SE. Means with different superscripts (^a,b,c,d^) between groups in the same column are significantly different at P < 0.05

### Alkaline comet assay in testes tissues

**Fig. 2:**
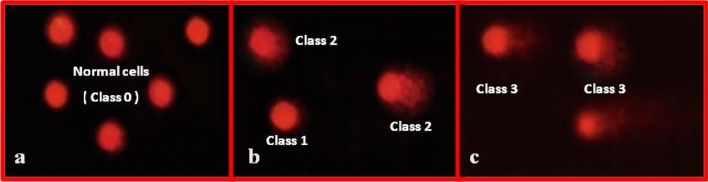
Visual score of normal DNA (class 0) **a**, cell with DNA damage (classes 1 and 2) **b**, cell with DNA damage (class 3) **c**, using comet assay in mice testes tissue collected from vinblastine treated groups

The DNA damage in the testes tissues of male mice exposed to a single dose of vinblastine (VB) at 3, 4.5, 6 and 10 mg/kg is summarized in (Table 2 and Fig. 2). Group of mice exposed to the low dose of VB (3 mg) showed relatively similar rate of DNA damage compared to control group (7.83 vs 7.21% respectively). The values of DNA damage were increased significantly in other doses of VB compared to control group. Dose-related relationship was recorded where the percentage of DNA damage reached 10.24, 16.61 and 21.43% after treatment with the doses 4.5, 6 and 10 mg/kg respectively. Additionally, the highest DNA damage of class 3 (tail longer than 2 × the diameter of the nucleus) was showed in mice exposed to 10 mg/kg VB.

### Chromosomal aberration analysis in mouse spermatocytes

(Table 3) illustrates the percentage of different types of CAs induced in mouse spermatocytes at diakinesis metaphase I after VB treatment. A significant and dose-dependent increase in the frequency of CAs was recorded. The maximum percentage of CAs (13.2%) was reached after single dose treatment at 10 mg/kg VB vs 3.80% for the control. Repeated dose treatment gave a higher incidence of CAs in comparing with the single dose treatment e.g. the dose 6 mg/kg induced 10.40 and 12.20% CAs after single and repeated treatment respectively. The majority of aberrations were in the form of univalent (X–Y and Autosomal univalent) with the superiority of X–Y univalent in comparison with autosomal univalent. The percentage of X–Y univalent for example reached 9.0% after treatment with a single dose of 10 mg/kg VB vs 3.40% for autosomal univalent.

**Table 3:** Frequency of chromosomal aberrations induced in mouse spermatocytes after treatment with vinblastine

Treatment and doses	Total abnormal metaphases		No and (%) of metaphases with different types of chromosome aberration					
	No	Mean(%) ± SE	X–Y univalent	Autosomal univalent	X–y u + A-u	Fragment and/or Break	Chain IV	Polyp
I. Control (Non-treated)	19	3.80 ± 0.37^a^	17(3.40)	2(0.40)	–	–	–	–
II. Vinblastine a-Single dose								
3 mg/kg	40	8.0 ± 0.77^b^	25(5.0)	12(2.40)	–	1(0.20)	–	2(0.40)
4.5 mg/kg	44	8.80 ± 0.37^b,c^	28(5.60)	12(2.40)	–	1(0.20)	–	3(0.60)
6 mg/kg	52	10.40 ± 0.93^c,d^	23(4.60)	20(4.0)	1(0.20)	3(0.60)	1(0.20)	4(0.80)
10 mg/kg	66	13.20 ± 1.16^e^	45(9.0)	17(3.40)	2(0.40)	2(0.20)	–	–
b-3 successive dose treatment								
3 mg/kg	51	10.20 ± 0.86^b,c,d^	27(5.40)	18(3.60)	–	4(0.40)	1(0.20)	1(1.0)
4.5 mg/kg	55	11.0 ± 0.83^c,d,e^	41(8.20)	8(1.60)	–	5(1.0)	–	1(1.0)
6 mg/kg	61	12.20 ± 0.37^d,e^	30(6.0)	18(3.60)	–	6(1.20)	–	7(1.40)

A total of 500 cells were analyzed (five mice per group; 100 cells/mouse). *X–Y u* X–Y univalent, *A-u* Autosomal univalent, *Polyp* Polyploidy. One way ANOVA–Tukey’s multiple comparisons test was used. The values having different superscript letters in each column are significantly different from one another

### Morphological sperm abnormalities

**Fig. 3:**
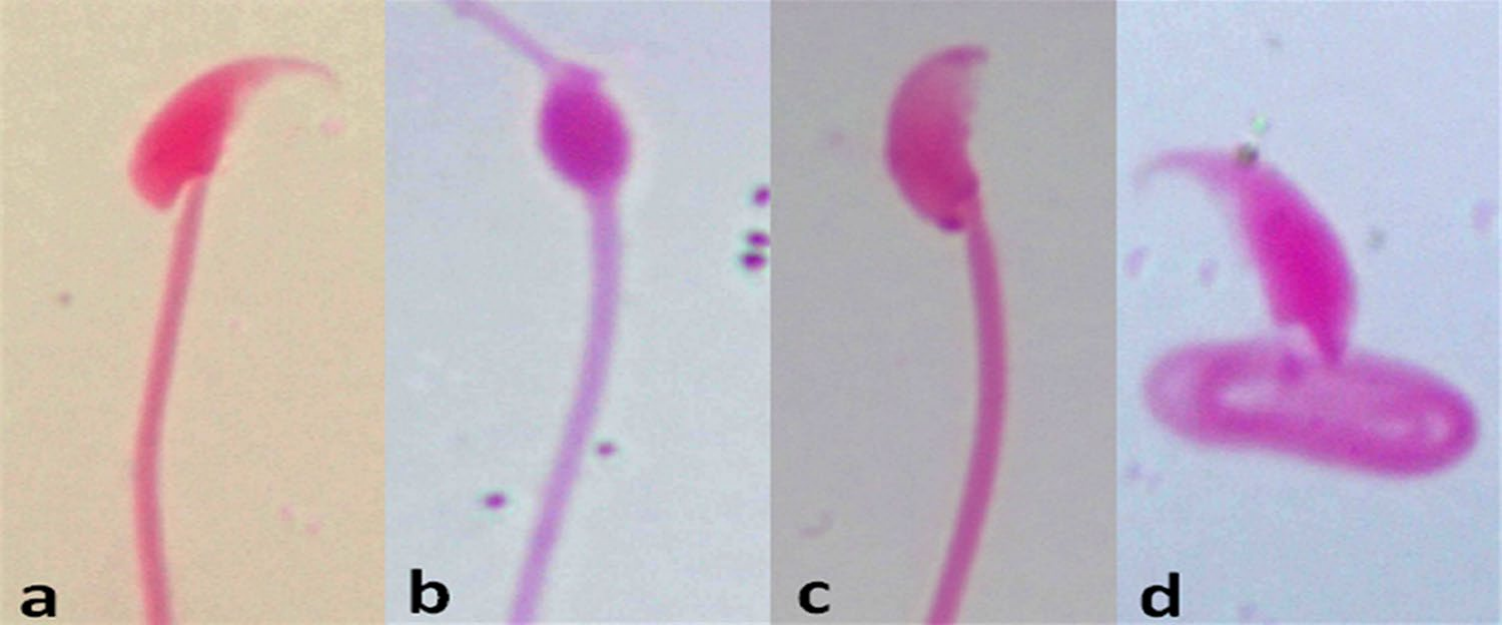
Sperm abnormalities showing **a** control, **b** amorphous head, **c**: head without hook, **d** coiled tail

The results in (Table 4; Fig. 3) demonstrated a significant (p < 0.05) and dose-dependent increase in the frequency of sperm abnormalities in comparison with the negative control. The maximum percentage of sperm defects reached 11.06% after treatment with the highest tested dose of 6 mg/kg vs 3.04% for the control. With respect to the types of sperm abnormalities, Coiled tail sperm represents the major percentage. Shapeless head sperm and head without hook were also recorded. In the highest tested dose, sperm tail defects (Coiled tail abnormality) represent 7.66% while the head abnormalities represent 3.4% of the total counted sperm (including different types of head abnormalities).

**Table 4:** .Percentage of sperm abnormalities induced in male mice after treatment with vinblastine

Experimental groups and doses	Total abnormal sperm		No. and (%)of different types of sperm abnormalities				
			Head abnormalities				Tail abnormalities
	No.	Mean (%) ± SE	Amorphous	Without hook	Triangle	Banana	Coiled tail
I-Control (−ve)	152	3.04 ± 0.19^a^	16(0.32)	4(0.08)	2(0.04)	1(0.02)	20(0.40)
II-Vinblastine 3 mg/kg	472	9.44 ± 0.13^b^	71(1.42)	69(1.38)	18(0.36)	23(0.46)	291(5.82)
4.5 mg/kg	520	10.40 ± 0.73^b,c^	85(1.70)	67(1.34)	19(0.38)	19(0.38)	330(6.60)
6 mg/kg	553	11.06 ± 0.14^c^	54(1.08)	87(1.74)	18(0.36)	11(0.22)	383(7.66)

Total number of examined sperms 5000 per each treatment (1000/mouse, five mice/group).One way ANOVA–Tukey’s multiple comparisons test was used. The values having different superscript letters in each column are significantly different from one another at p < 0.05

## Discussion

The majority of chemotherapies have no selectivity to cancer cells and affect healthy body tissues where the cells are constantly growing and dividing. However, in cancer survivors, a second malignancy frequently occurs after chemotherapy, which warrants detailed genotoxicity testing of the chemotherapeutic agents [5, 10]. Herein, a comprehensive study was performed to evaluate the genotoxic effects of vinblastine (VB) using several endpoints as biomarkers. The results showed that VB induced a significant percentage of chromosomal aberrations in somatic cells (bone marrow) which was dose-dependent in both single and repeated treatments. The frequency of chromosomal aberrations reached about 4-folds increase after treatment with a single dose of 10 mg/kg and about 6-folds increase after three successive doses at 6 mg/kg of VB as compared with the negative control. A pronounced number of polyploidy was recorded at all doses and also a number of metaphases with fragments (numerical and structural aberrations). Vinca alkaloids appear to exert their antitumor effect by binding to tubulin proteins within the cells. Because these proteins are essential contractile proteins of the mitotic spindle of dividing cells, this binding leads to mitotic arrest [6, 7] which may explain the appearance of a high percentage of polyploidy after VB treatment. Generally, in the absence of an intact mitotic spindle, the chromosomes may clump in an unusual grouping or may disperse throughout the cytoplasm [22]. Previous studies with mouse bone marrow cells revealed the presence of aneuploidy and polyploidy after VB treatment [11, 23]. Liang et al. [11] demonstrated that the injection of mice with the dose 9 mg/kg induced a high percentage of polyploidy (ranging from 1.5 to 5.2%) in all of the 10 treated mice sampled at 17–96 h after injection. Such results are greatly support the results obtained in the present work. Due to their action as mitotic spindle poisons, vinca alkaloids are regarded as aneugens [24]. Vinca alkaloids are also cell cycle phase-specific ‘S and M’ phase poisons. The present study also demonstrated DNA damage in spleen cells which also recorded a dose-dependent relationship. The effect of VB in somatic cells is coincided well with the previous results of other authors: micronuclei and chromosomal aberrations were significantly induced in mouse bone marrow after treatment with VB [4, 25, 26] and in cultured human lymphocytes after VC treatment [27]. However, the induction of micronuclei may reflect chromosomal damage or loss of whole chromosomes from the daughter nuclei. Oxidative DNA damage in cultured human lymphocytes and an increase in the cells mitotic index (*p* < 0.05) were also demonstrated after VB and VC treatment [2]. They also induced chromosomal mutations in in vivo and in cultured cancer cells [28, 29]. Moreover, VB was found to affect nucleic acids, amino acids, proteins and purine synthesis [30]. Tyagi et al. [31] made a spectroscopic study which showed that plant alkaloid binds with DNA via A-T and G-C base pairs along with the phosphate backbone of the helix. In addition, the docking studies have indicated an interaction of VB with the adenine base of DNA helix via hydrogen bonding. Conversely, other studies showed lack of mutagenic activity of Vinca alkaloids [32, 33]. Generally, aneugenic agents are expected to have a low level of clastogencity as a result of their poisoning effect on the mitotic spindle [34].

Chromosomal aberration analysis was also examined in mouse spermatocytes. The current results indicated a significant percentage of chromosomal aberrations (p ˂ 0.05) after single and repeated treatments with the presence of dose-relationship. The maximum percentage of aberrations reached approximately 3.5 and 3.2-folds increase as compared to the negative control after single dose of 10 mg/kg and repeated doses of 6 mg/kg VB respectively. The majority of aberrations were X–Y and Autosomal univalent (gonosomal univalency) and fragments. Induction of significant percentages of aberrant primary spermatocytes (P ≤ 0.01) was also recorded by Palo et al. [8] with atypical bivalents. Moreover, the current results indicated DNA damage in the testes as measured by alkaline comet assay and evidenced by comet tail length. There is a great correlation between DNA damage result and chromosomal aberrations in the testes and each one support the other. Such effect may be as a result of oxidative stress exerted by VB in the form of reactive oxygen and nitrogen species (ROS/RNS) in germ cells and to its depleted activity on the antioxidant enzymes: SOD, CAT and GPx. VB was also reported to induce a disturbance in the intracellular mediator’s ‘H_2_O_2_ and Ca^2+^level’ [2, 35]. In addition, it was demonstrated to alter calcium homeostasis via mitochondrial membranes leading to cytotoxicity and chromosome instability. Inducing of apoptosis is considered one of its mechanisms of action. The present results are also concerned with measuring the morphological sperm abnormalities after VB treatment (at different doses). Different categories of abnormal sperm were noticed and indicated the transmission of VB-induced cytogenotoxic effects from spermatocyte to sperm. Jagetia et al. [36] demonstrated that different doses of VB can affect mouse spermatogenesis as measured by DNA flow cytometry. Previous reports also showed significant percentages of aberrant spermatogonial metaphases and chromosomal aberrations in VB-treated mice. VB also significantly increased the percentages of aberrant primary spermatocytes and the morphological sperm defects as indicated by Palo et al. [8] Also AL-Zubaidi [9] demonstrated that high doses (4, 5 and 6 mg/kg) of vinblastine sulphate have negative effects on sperm parameters and histological feature of the testis in mice. These result in addition to the results of the current work emphasizes the ability of VB to cause genetic defects that may be passed on to future generations. The cancer survivors who are treated with chemotherapy before or during their reproductive years have faced serious problem. Such effect must be taken into consideration when choosing the chemotherapeutic regimens which may jeopardize the genetic health of offspring [37]. Infertility is another risk problem that faces cancer survivors. Therapeutic modalities such as radiation therapy and chemotherapy are highly effective in treating cancer, but their gonadotoxic side effects can severely impair fertility in an agent- and dose-dependent way. Spermatogenesis for example in long-term cancer survivors has elucidated evidence of persistent azoospermia or severe oligozoospermia in up to 24% of patients [38]. This effect warrants several years after chemotherapy to recover. The alkylating agents such as cyclophosphamide and isophosphamide caused permanent azoospermia in 80–90% of cases [39]. Azoospermia was detected follow the use of VB in testicular cancer patients and it may be reversible within 2–3 years [40]. In the present study VB induced a high percentage of sperm defects (tail and head defects) with a pronounced appearance of coiled tail sperm. Also the absence of acrosome and amorphous or misshape head sperm were recorded. These defects might affect the ability of the sperm to reach and penetrate an egg. Coiling of the sperm tail may limit or stop its motility and also acrosome plays a crucial role in its function. Sperm motility and acrosome reaction are considered key functions in the control of reproduction and also they are essential for spermatozoa to become able to fertilize the egg [41, 42]. The sperm morphology was reported to be genetically controlled by numerous autosomal and sex-linked genes and the formation of a normal sperm head or tail involves intricate synchronous morphological and biochemical steps [43]. Oxidative stress, high levels of lipid peroxidation and oxidative DNA damage accompanied by mutation were considered the major causes of male infertility. Mutations that influence sperm quality include conditions that affect the morphological appearance of spermatozoa and their competence of fertilization [44]. The current results emphasize the positive correlation between cytogenetic damage (CAs & DNA damage) in germ cells and sperm abnormalities.

## Conclusion

The present work demonstrated that vinblastine has a genotoxic effect evidenced by DNA damage and chromosomal aberrations (CAs). In bone marrow cells CA analysis revealed a pronounced number of polyploidy metaphases which reflect the effect of VB in the mitotic spindle. CAs induced by VB in spermatocytes were transmitted to sperm causing several defect categories. Vinblastine especially affects the motility of the sperm. Sperm defects are expected to affect the fertility of the cancer survivors or might be result in malformation in the future offspring. The results of the present work must be taken into consideration while using VB in the chemotherapeutic regimens. All of the data obtained are very important and provided a detailed study on the genotoxicity of vinblastine; when published, they will be very useful for patients, doctors, and pharmaceutical companies in determining the safe use of VB chemotherapy.

**Future directions**: In the future, we may involve other studies eg., dominant lethal mutation, genes controlling sperm maturation using real-time PCR analysis, and so on…
